# Evaluating photodynamic therapy versus brolucizumab as a second-line treatment for polypoidal choroidal vasculopathy

**DOI:** 10.1186/s40942-024-00553-5

**Published:** 2024-04-08

**Authors:** Ryoh Funatsu, Hiroto Terasaki, Naohisa Mihara, Shozo Sonoda, Hideki Shiihara, Taiji Sakamoto

**Affiliations:** Department of Ophthalmology, Kagoshima University Graduate School of Medical and Dental Sciences, Kagoshima, Japan

**Keywords:** Polypoidal choroidal vasculopathy, Photodynamic therapy, Brolucizumab, Neovascular age-related macular degeneration

## Abstract

**Background:**

To compare the one-year outcomes between intravitreal brolucizumab (IVBr) monotherapy and photodynamic therapy (PDT) as a second-line treatment in patients with polypoidal choroidal vasculopathy (PCV) who did not respond to first-line therapy.

**Methods:**

This case–control study included eyes with PCV that do not respond to aflibercept or ranibizumab. The patients were retrospectively registered. We compared outcomes, including best-corrected visual acuity (BCVA), anatomical results, and the need for additional treatments, between IVBr and a combination therapy using PDT as second-line treatments for refractory PCV, after adjusting for potential confounders. We analyzed E-values to evaluate the robustness of the results against unmeasured confounders.

**Results:**

Twenty-two eyes received IVBr, and twenty-four underwent PDT. No apparent differences were observed in BCVA and central macular thickness (CMT) changes from baseline between the groups (IVBr vs. PDT: BCVA, 0.01 ± 0.47 logMAR vs. 0.04 ± 0.18 logMAR, P-value = 0.756; CMT: − 36.3 ± 99.4 μm vs. − 114.7 ± 181.4 μm, P-value = 0.146). Only in the PDT group, five eyes (20.8%) did not require additional treatment after the second-line treatment, the adjusted odds ratio indicating no further treatment needed was 11.98 (95% confidence interval: 1.42–2070.07, P-value = 0.019). The E-value for the adjusted odds ratio was 23.44.

**Conclusions:**

Both second-line treatments for PCV exhibited similar visual and anatomical outcomes. Only in the PDT-treated eyes were there some patients who did not require further treatment after second-line therapy.

**Supplementary Information:**

The online version contains supplementary material available at 10.1186/s40942-024-00553-5.

## Background

Neovascular Age-related macular degeneration (nAMD) is a disease characterized by macular abnormalities and neovascularization that frequently results in severe vision impairment [1, 2], and is one of the leading causes of blindness worldwide [3]. Managing polypoidal choroidal vasculopathy (PCV), a specific nAMD subtype, is complex, as it can cause both a gradual decline in vision due to retinal damage from exudative changes, and a rapid and significant vision loss upon the rupture of polypoidal lesions [4, 5]. Current first-line treatments for PCV generally include anti-vascular endothelial growth factor (VEGF) drugs and photodynamic therapy (PDT) [6–8]. Due to the requirement for specialized equipment for PDT and the need for patients to avoid direct sunlight for a period following the procedure [9], anti-VEGF therapy is often preferred as the primary treatment in primary care environments.

However, resistance to anti-VEGF therapy is observed in certain cases of PCV [10–12]. These cases are associated with suboptimal functional outcomes due to recurrent and persistent exudative changes, demanding frequent and semi-permanent administration of expensive anti-VEGF drugs. Identifying efficacious second-line treatments is essential for patients unresponsive to anti-VEGF therapy, with the goal of preserving vision and alleviating the financial impact on both individuals and society [13]. The standard approach to treating refractory nAMD at present includes switching to other anti-VEGF medications and combination therapies utilizing PDT [10–12, 14–17]. Brolucizumab, recently approved, exhibits considerable effectiveness when employed as second-line treatment for nAMD [14, 15]. In comparison, PDT outperformed anti-VEGF drugs monotherapy in treatment-naïve eyes with PCV, and also showed potential benefits for patients resistant to anti-VEGF therapy [7, 10–12, 18]. Both treatments would serve as effective second-line treatments for PCV. However, determining the relative effectiveness of intravitreal brolucizumab compared to PDT for PCV that does not respond to aflibercept or ranibizumab requires further investigation.

This study is designed to evaluate the treatment outcomes of brolucizumab versus PDT for PCV as a second-line treatment, employing methods to address bias, with the goal of determining a more effective second-line treatment strategy for PCV.

## Methods

This study was conducted at Kagoshima University Hospital and received ethical approval from the Ethics Committee of Kagoshima University, Japan (Approval No. 16012). All procedures followed the tenets of the Declaration of Helsinki.

## Study design

This study compared the efficacy of PDT to brolucizumab monotherapy as a second-line treatment for patients with PCV who did not respond to first-line anti-VEGF agents (aflibercept or ranibizumab). We conducted a retrospective case–control study on patients with PCV treated at the Department of Ophthalmology, Kagoshima University Hospital, from January 2014 to October 2022.

**Fig. 1:**
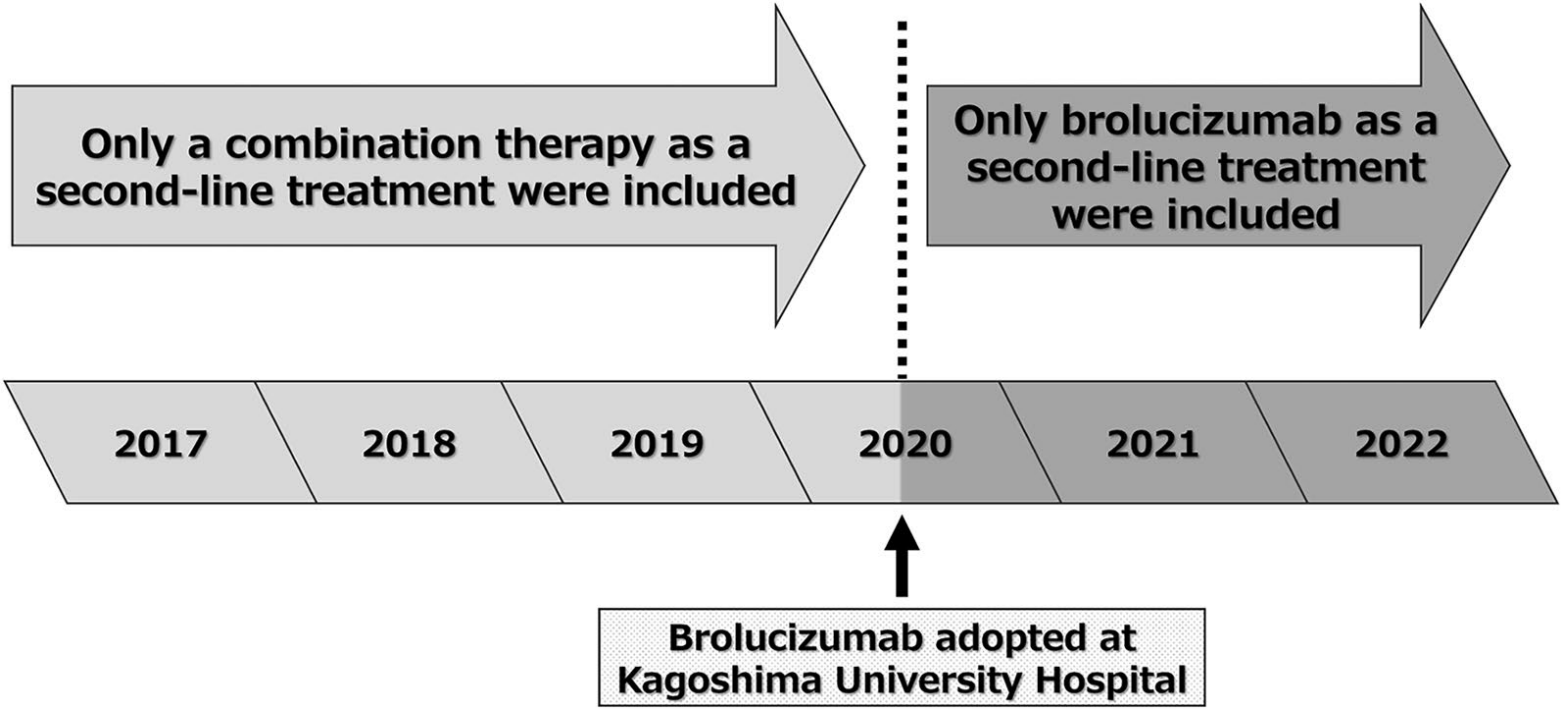
Inclusion criteria for each of the treatments: a study design overview

Selection bias, possibly arising from unmeasurable confounders such as physicians’ preferences, may influence the choice between combination therapy and brolucizumab monotherapy in retrospective comparisons of outcomes [19]. To mitigate selection bias, we restricted our analysis of PDT cases to those treated before June 2020, when brolucizumab was adopted at our hospital. Consequently, this limited some of the dataset to a period when the choice between PDT and brolucizumab monotherapy did not occur (Fig. 1).

## Inclusion and exclusion criteria

Two retina specialists (R.F., N.M.) diagnosed PCV at the initial examination by the following criteria based on previous reports, with a third specialist (H.T.) making the final decision in cases of disagreement: (1) age 50 or older, (2) color fundus photograph (CFP) using DRI OCT Triton (Topcon, Tokyo, Japan) showing an orange nodule or hemorrhagic pigment epithelial detachment (PED), (3) indocyanine green angiography (ICGA) using Spectralis (Heidelberg Engineering, Heidelberg, Germany) showing polypoidal dilation, (4) spectral domain-optical coherence tomography (SD-OCT) using Spectralis showing multiple PED or sharply peaked PED, and (5) fluorescein angiography using Spectralis or ICGA showing macular neovascularization [20, 21]. We defined eyes as treated with first-line anti-VEGF therapy if they received monthly injections of aflibercept, ranibizumab, or both for three consecutive months (induction therapy), without prior treatment. For eyes to be classified as refractory to anti-VEGF therapy, they must meet at least one of these criteria: (1) Subretinal hemorrhage (SRH) development, PED enlargement, or persistent retinal fluid presence a month after an injection after the induction phase or in the six months before switching treatments in the maintenance phase; (2) Eyes with an average injection interval of 8 weeks or less between the last three injections before moving to second-line treatments, excluding the induction therapy; (3) Eyes unable to maintain a 'dry' state with 8-week interval injections in the six months before transitioning to second-line treatments. The exclusion criteria for the study were as follows: (1) Eyes previously treated with PDT, macular area photocoagulation, or intravitreal brolucizumab injections; (2) Eyes with a history of ruptured polypoidal lesions; (3) Eyes not monitored for over 12 months post-treatment switch; (4) Eyes with other intraocular diseases that impair vision; (5) Eyes with unclear imaging results; (6) Eyes with a history of intraocular surgeries, excluding cataract surgery; (7) Eyes with retinal pigment epithelial tears involving the fovea; (8) Cases in which the last dose of the anti-VEGF drug was administered more than 4 weeks before PDT. If both eyes of a patient met these criteria, only the eye treated later was included in the study.

## Second-line treatments

For this study, as second-line treatment, cases using brolucizumab were defined as the brolucizumab group and cases using PDT were defined as the PDT group. A combination therapy is defined as a treatment in which an eye receives anti-VEGF therapy (either aflibercept or ranibizumab) within one week prior to PDT [22]. Therefore, the PDT group comprises cases with combination therapy and cases where the last dose of the anti-VEGF drug was administered more than one week but less than or equal to four weeks prior. For the PDT group, standard PDT was performed for all cases. The size and placement of PDT irradiation were guided by ICGA findings to encompass the full extent of the largest lesion diameter [7]. The decision regarding second-line treatment and PDT irradiation parameters was made by the treating physician. Following the second-line treatment, anti-VEGF therapy and PDT were administered on an as-needed (pro re nata) basis, with careful monitoring for signs of exudative activity. The scheduling of treatments and the choice of anti-VEGF agents were at the discretion of the individual physicians.

## Outcomes

The main outcome of this study is the change in best-corrected visual acuity (BCVA) observed 12 months after transitioning to the second-line treatment for nAMD. The secondary outcomes of the study were central macular thickness (CMT), the total count of injections, the percentage of patients who avoided intravitreal injections, and the incidence of adverse ocular complications in the first year after the second-line treatment. Intravitreal injections administered as part of the combination therapy were not included in the total count of injections prior to the second-line treatment. Furthermore, since cases requiring PDT often necessitate hospitalization and the scheduling of treatment depends on factors beyond the primary physician's control, the interval between the last injection prior to starting the combination therapy and the initiation of the combination therapy itself was not included in the count.

Subfoveal choroidal thickness (SFCT) was measured manually in a vertical direction, selecting the deepest point of the foveal depression using enhanced depth imaging-OCT (Spectralis, in a five-line raster scan mode, 30°, with image averaging set at 100). Similarly, the size of the largest polyp was manually measured in a vertical direction by selecting the tallest point at the location of the polypoidal lesion in ICGA, using OCT imaging performed in volume scan mode (30 × 25°, centered on the fovea, horizontal, with image averaging set at 100). CMT was defined as the average thickness within a 1 mm diameter area centered on the fovea. For cases receiving combination therapy, OCT findings from the day of anti-VEGF drug administration were utilized. FA and ICGA images taken closest to the start of the second-line treatment were chosen for analysis. Fibrotic scar, SRH, subretinal fluid (SRF), and intraretinal fluid (IRF) were identified using CFP, FA, and OCT images at the second-line treatment. These findings were evaluated through joint consultation by two retina specialists, R.F. and N.M based on previous report, with a third specialist (H.T.) making the final decision in cases of disagreement. [23]

## Potential confounders

According to previous reports, factors related to visual prognosis in PCV include pretreatment BCVA, intraretinal fluid, SRH, choroidal thickness, choroidal vascular hyperpermeability, and the size of the largest polyp [23–26]. Furthermore, SRH and vitreous hemorrhage are recognized as complications arising from PDT [7, 27, 28]. These factors are associated with treatment outcomes and may influence physicians' choice of second-line treatments, potentially serving as confounders. Based on these previous reports, we created a directed acyclic graph, and this graph served as a reference for identifying potential confounders that needed adjustment (**Figure S1**). [29]

## Statistical analysis

We compared patient characteristics and outcomes. For quantitative variables, comparisons were made using the Mann–Whitney U test, while qualitative variables were evaluated using logistic regression analysis. To adjust the confounders, we conducted multivariable linear regression analysis or multivariable logistic regression analysis using the identified potential confounders as independent variables. Similarly, a model was analyzed to include items demonstrating statistically significant patient characteristics differences. In this report, we performed logistic regression analyses with Firth's bias reduction method due to quasi-complete separation [30, 31]. The statistical cutoff value was set at *P*-value = 0.05. We also examined E-values to conduct a sensitivity analysis for unmeasured confounders [32]. The E-value is an indicator that quantifies the degree to which unmeasured confounders must be associated with both the treatment selection and the outcome to nullify the results, essentially measuring the necessary extent of bias to change the conclusions. We used DAGitty to create a directed acyclic graph [33], and all analyses were performed using the R software (version 4.3.0).

## Results

### Patient characteristics

In this study, brolucizumab was administered as a second-line treatment to 22 patients (22 eyes), while 24 patients (24 eyes) received PDT. In the PDT group, 19 eyes (79.2%) underwent combination therapy, and all eyes that did not receive combination therapy were treated with anti-VEGF drugs within four weeks prior to PDT. Table 1 presents the details of patient characteristics for each group. The brolucizumab group had an average of 22.6 ± 14.6 previous injections, significantly higher than the PDT group's average of 11.9 ± 6.9 (P-value = 0.009). The presence of IRF at the time of second-line treatment was higher in the brolucizumab group compared to the PDT group (59.1% vs. 29.2%, P-value = 0.044). No apparent differences were observed in other pre-treatment factors (All P-values ≥ 0.113, Table 1).

**Table 1:** Comparisons of patient characteristics between those treated with brolucizmab and photodynamic therapy as second-line treatments

Characteristic	Brolucizmab, N = 22	Photodynamic therapy, N = 24	P-values
Age (years)	76.6 ± 6.8; 78.0	74.7 ± 8.2; 76.0	0.415
Female	4 (18.2%)	4 (16.7%)	0.890
Total number of previous injections	22.6 ± 14.6; 20.0	11.9 ± 6.9; 11.0	0.009
BCVA (logMAR)	0.48 ± 0.52; 0.35	0.30 ± 0.33; 0.30	0.314
SRF	18 (81.8%)	23 (95.8%)	0.149
IRF	13 (59.1%)	7 (29.2%)	0.044
CVH	18 (81.8%)	16 (66.7%)	0.253
The maximum PED size (μm)	423.1 ± 240.4; 405.5	422.3 ± 280.2; 330.0	0.750
SRH	8 (36.4%)	8 (33.3%)	0.831
Fibrotic scar	11 (50.0%)	8 (33.3%)	0.261
CCT (μm)	216.0 ± 143.8; 152.5	244.4 ± 77.1; 234.5	0.113
CMT (μm)	331.7 ± 110.9; 304.5	389.1 ± 174.2; 343.0	0.202
The mean of previous treatment intervals (weeks)	7.6 ± 3.1; 7.7	6.4 ± 2.3; 5.7	0.319

The mean ± standard deviation; median was used for continuous variables, and numbers and percentages were used as nominal variables. P-values were calculated using logistic regression analysis using Firth’s bias reduction method for qualitative variables and the Mann–Whitney U test for continuous variables. *BCVA* best-corrected visual acuity, *SRF* subretinal fluid, *IRF* intraretinal fluid, *CVH* choroidal vascular hyperpermeability, *PED* pigment epithelial detachment, *SRH* subretinal hemorrhage, *CCT* central choroidal thickness, *CMT* central macular thickness

## Main outcome

**Fig. 2:**
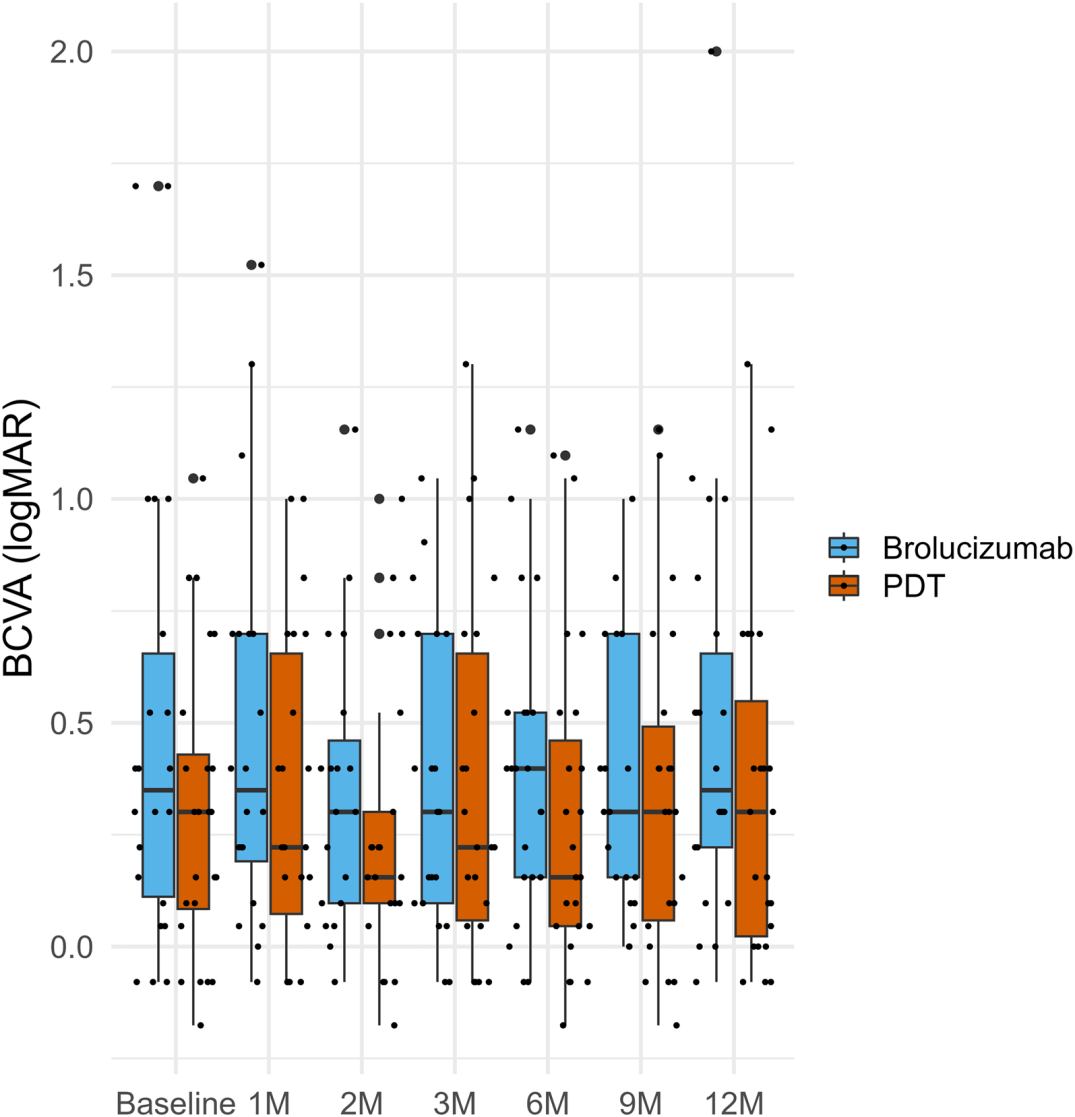
The box-and-whisker plots for the mean BCVA changes after second-line treatments. BCVA, best-corrected visual acuity

Twelve months after second-line treatment, BCVA was not apparently different between groups, with a mean of 0.49 ± 0.47 logMAR for the brolucizumab group and 0.34 ± 0.39 logMAR for the PDT group (P-value = 0.236, Table 2 and Fig. 2). At all other time points, including baseline, no significant differences in BCVA were observed between the groups (brolucizumab vs. PDT: baseline, 0.48 ± 0.52 logMAR vs. 0.30 ± 0.33 logMAR, P-value = 0.314; 3 months, 0.36 ± 0.33 logMAR vs. 0.37 ± 0.40 logMAR, P-value = 0.817; 6 months, 0.39 ± 0.33 logMAR vs. 0.28 ± 0.35 logMAR, P-value = 0.186, Table S1). No apparent differences were observed in BCVA changes from baseline to 12 months within either group (brolucizumab, P-value = 0.465; PDT, P-value = 0.330, **Table S1**). Similarly, there were no significant differences in BCVA changes at 12 months from baseline between the groups (brolucizumab vs. PDT: 0.01 ± 0.47 logMAR vs. 0.04 ± 0.18 logMAR, P-value = 0.756).

**Table 2:** Comparison of twelve-month outcomes in patients who switched to brolucizmab or photodynamic therapy

Characteristic	Brolucizmab, N = 22	Photodynamic therapy, N = 24	P-values
BCVA (logMAR)	0.49 ± 0.47; 0.35	0.34 ± 0.39; 0.30	0.236
The change of BCVA from the baseline (logMAR)	0.01 ± 0.47; 0.02	0.04 ± 0.18; 0.00	0.756
CMT (μm)	295.4 ± 93.9; 267.0	280.4 ± 69.2; 256.0	0.742
The change of CMT from the baseline (μm)	− 36.3 ± 99.4; − 22.5	− 114.7 ± 181.4; − 61.0	0.146
The total number of injections	4.9 ± 1.6; 5.0	4.5 ± 3.3; 4.5	0.482
The number of additional treatment-free cases	0 (0.0%)	5 (20.8%)	0.025

The mean ± standard deviation; median was used for continuous variables, and numbers and percentages were used as nominal variables. P-values were calculated using logistic regression analysis using Firth’s bias reduction method for qualitative variables and the Mann–Whitney U test for continuous variables. **Abbreviations**: BCVA, best-corrected visual acuity; CMT, central macular thickness

## Secondary outcomes

There was no apparent difference in CMT at 12 months after second-line treatment, averaging 295.4 ± 93.9 μm in the brolucizumab group and 280.4 ± 69.2 μm in the PDT group (P-value = 0.742, Table 2). There was also no difference in CMT change from baseline at 12 months between the groups (brolucizumab vs. PDT: -36.3 ± 99.4 μm vs. -114.7 ± 181.4 μm, P-value = 0.146). In the 12 months following second-line treatment, the average total number of injections was 4.9 ± 1.6 in the brolucizumab group and 4.5 ± 3.3 in the PDT group, indicating no significant difference (P-value = 0.482). However, five eyes (20.8%) only in the PDT group did not require additional treatment, which was statistically significant (P-value = 0.025).

**Fig. 3:**
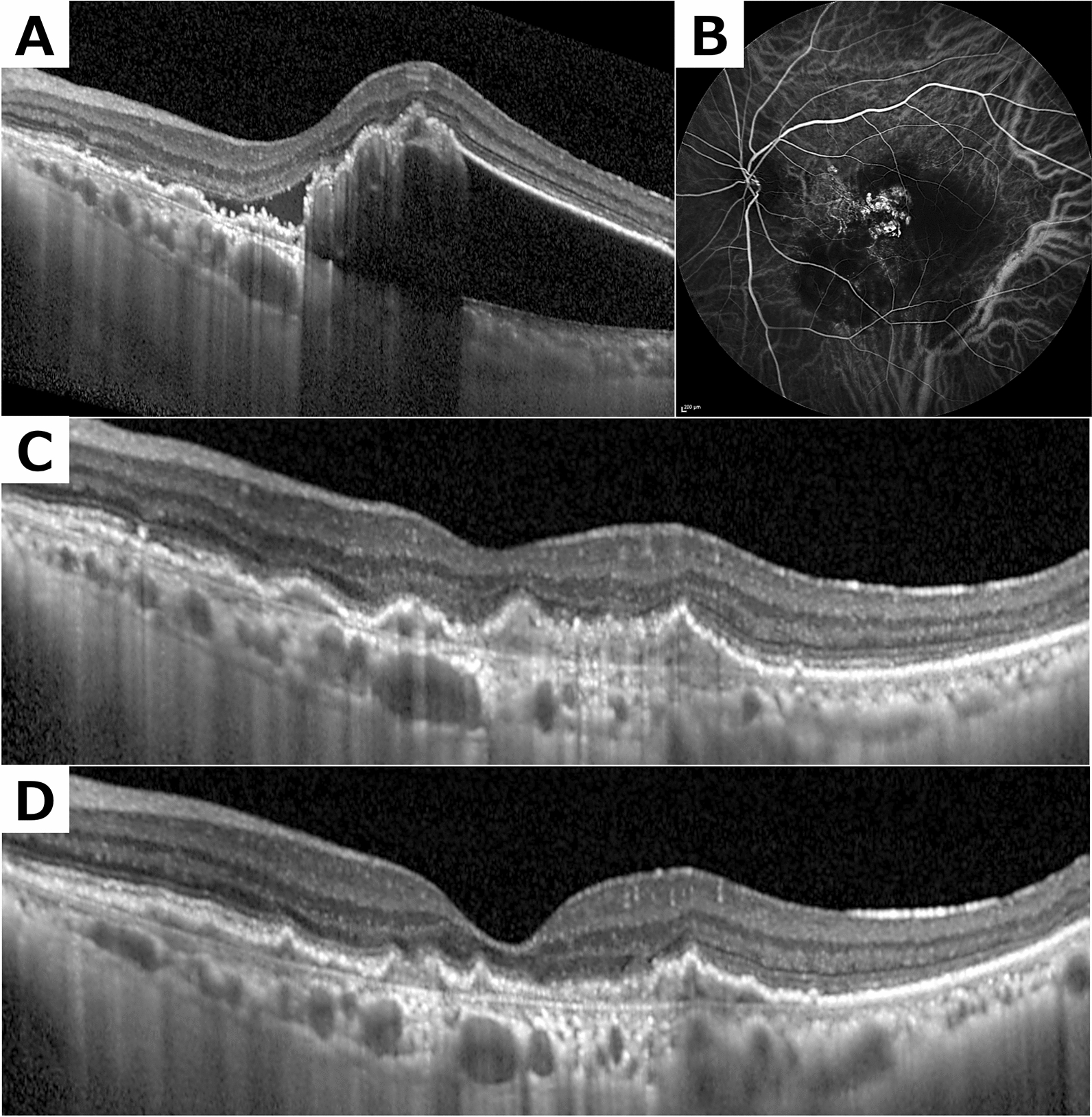
A polypoidal choroidal vasculopathy case managed with secondary PDT without additional treatment**. A** Pre-treatment Optical coherence tomography (OCT) reveals pigment epithelial detachment (PED) with sharp peaks and sub-retinal pigment epithelium ring-like lesions. **B** Corresponding indocyanine green angiography (ICGA) captures multiple polyps. **C** 12-month post-PDT OCT shows diminished PED and resolved retinal fluid with no further treatment. **D** A similar OCT of the subfoveal area indicates remission of retinal fluid. *PDT* photodynamic therapy, *OCT* optical coherence tomography, *PED* pigment epithelial detachment; *ICGA* indocyanine green angiography

For the PDT group, the crude odds ratio (OR) indicating no further treatment needed after second-line therapy was 12.69 (95% confidence interval: 1.30–1706.23, Table S2). Based on the model derived from Figure S1, the adjusted odds ratio (OR) was 11.98 (95% confidence interval: 1.42–2070.07, P-value = 0.019, Table S2). When this model was extended to include the total number of previous injections, the adjusted OR was 95.38 (95% confidence interval: 1.48–5.76 × 10^11, P-value = 0.023). The E-value for the adjusted odds ratio (OR), derived from the model based on Figure S1, was 23.44. Figure 3 presents a representative case where no additional treatment was required after second-line therapy.

## Sub-analysis on additional treatments after PDT

Patients not requiring additional treatment for one year following second-line therapy showed a significantly thinner CMT at 12 months and older than those needing further treatment (CMT at 12 months: 216.0 ± 23.3 μm vs. 298.3 ± 67.2 μm, P-value = 0.001; age: 81.4 ± 6.8 years vs. 73.0 ± 7.7 years, P-value = 0.042, Table 3). Other factors showed no clear differences between the two groups (All P-values ≥ 0.095).

**Table 3:** Comparing characteristics based on the additional treatment needs after second-line treatments in the PDT group

Characteristic	Non-injection free cases, N = 19	Injection-free cases, N = 5	P-values
At the second-line treatment			
Age (years)	73.0 ± 7.7; 73.0	81.4 ± 6.8; 82.0	0.042
Female	4 (21.1%)	0 (0.00%)	0.479
Total number of previous injections	12.1 ± 7.6; 11.0	11.2 ± 3.7; 11.0	0.943
BCVA (logMAR)	0.29 ± 0.35; 0.30	0.35 ± 0.29; 0.30	0.591
SRF	18 (94.7%)	5 (100%)	0.242
IRF	4 (21.1%)	3 (60.0%)	0.683
CVH	11 (57.9%)	5 (100%)	0.747
The maximum PED size (μm)	399.6 ± 291.7; 297.0	508.6 ± 238.5; 483.0	0.213
SRH	7 (36.8%)	1 (20.0%)	0.747
Fibrotic scar	6 (31.6%)	2 (40.0%)	0.747
CCT (μm)	244.7 ± 85.6; 233.0	243.2 ± 35.3; 236.0	0.915
CMT (μm)	396.8 ± 156.2; 353.0	360.0 ± 251.8; 246.0	0.095
The mean of previous treatment intervals (weeks)	6.3 ± 2.4; 5.7	6.7 ± 1.5; 6.7	0.595
Twelve months after a second-line treatment			
BCVA (logMAR)	0.35 ± 0.42; 0.23	0.31 ± 0.29; 0.40	0.881
CMT (μm)	298.3 ± 67.2; 276.5	216.0 ± 23.3; 215.0	0.001
The change of BCVA from the baseline (logMAR)	0.06 ± 0.15; 0.00	− 0.05 ± 0.27; − 0.05	0.318
The change of CMT from the baseline (μm)	− 106.6 ± 168.3; -67.5	− 144.0 ± 243.5; -31.0	0.852

The mean ± standard deviation; median was used for continuous variables, and numbers and percentages were used as nominal variables. P-values were calculated using logistic regression analysis using Firth’s bias reduction method for qualitative variables and the Mann–Whitney U test for continuous variables. *PDT* photodynamic therapy, *BCVA* best-corrected visual acuity, *SRF* subretinal fluid; IRF, intraretinal fluid, *CVH* choroidal vascular hyperpermeability, *PED* pigment epithelial detachment, *SRH* subretinal hemorrhage, *CCT* central choroidal thickness, *CMT* central macular thickness

## Complications

In the brolucizumab group, SRH developed in one eye (4.5%), and intraocular inflammation occurred in three eyes (13.6%) after the treatment switch. All three eyes with intraocular inflammation were switched back to anti-VEGF drugs before starting second-line treatment. Two of the three eyes exhibited inflammation confined to the anterior chamber, which resolved following treatment with betamethasone sodium phosphate eye drops. Their final BCVA was 0.30 logMAR and 0.70 logMAR, respectively. The remaining patient with vitreous opacity underwent treatment with betamethasone sodium phosphate eye drops and two sub-Tenon's capsule injections of triamcinolone acetonide in six weeks intervals. Following this treatment, the patient was diagnosed with steroid-induced glaucoma. Discontinuing the steroids led to the normalization of intraocular pressure, resulting in a final BCVA of 0.0 logMAR. In the PDT group, two eyes (8.3%) developed SRH with no other evident complications.

## Discussion

The current study compared the efficacy of second-line treatment on PCV between PDT and brolucizumab, employing a combination of study design and statistical methods to adjust for confounders as comprehensively as possible. The results indicated no significant differences in visual and anatomical outcomes between PDT and brolucizumab; however, only in the PDT group were cases requiring no additional treatment, with an adjusted OR of 11.98. Notably, the E value for this adjusted OR was 23.44, indicating that this association cannot be ruled out unless there are confounders associated with an OR of 23.44 for treatment selections and the incidence of additional treatment-free cases. Thus, it would be difficult to attribute the current findings solely to the bias inherent in observational studies.

The EVEREST II study, comparing the efficacy of ranibizumab monotherapy with combination therapy utilizing PDT as a first-line treatment for PCV, demonstrated more significant improvement in BCVA and greater reduction in CMT in the combination therapy group [7]. Additionally, the Fujisan study showed the effectiveness of a combination therapy as a second-line treatment [22]. These findings indicate that combination therapy with PDT might be more effective for treating PCV in both first- and second-line treatment settings. However, the PLANET study, which evaluated the efficacy of combination therapy as a second-line treatment in patients initially treated with aflibercept monotherapy, did not find any significant differences in treatment outcomes between the monotherapy and combination therapy groups [34]. Despite some controversy, compared to ranibizumab, aflibercept has demonstrated similar treatment outcomes with fewer doses in nAMD therapy [35]. Additionally, it may also be effective in treating nAMD cases that are unresponsive to ranibizumab [17]. A potential reason for the uncertain efficacy of PDT observed in the PLANET study could be the varying efficacies of the individual drugs. Furthermore, brolucizumab has demonstrated superior efficacy in achieving anatomical outcomes compared to other anti-VEGF drugs [36], along with significant effectiveness in treatment switching [14]. These findings indicate its potential as an effective treatment for nAMD that is resistant to other therapies. However, this study demonstrated that brolucizumab and PDT yielded comparable results in terms of both functional and anatomical outcomes when used as second-line treatments for PCV. The results align with those of the PLANET study, [34] indicating that brolucizumab may not offer significant differences in treating PCV that is resistant to ranibizumab or aflibercept. It should be noted that these results do not clarify whether brolucizumab or PDT is more effective as a first-line treatment.

Interestingly, the current study revealed that in the PDT group, 20.8% of cases required no additional treatment after second-line therapy, a finding not observed in the brolucizumab group. Compared to anti-VEGF monotherapy, PDT has consistently demonstrated its ability to reduce the number of injections required [7, 22]. The Fujisan study revealed that patients undergoing combination therapy utilizing PDT for PCV as a second-line treatment experienced an approximately 25% injection-free rate, similar to the current results [22]. Even with brolucizumab, supposed to be more effective against exudative changes compared to other anti-VEGF drugs, the current study found no patients who were completely free from the additional treatments. Given the individual patient's burden and the economic impact on society, minimizing the need for continuous anti-VEGF drug administration is crucial [13]. Therefore, the advantage of PDT in achieving injection-free status for patients resistant to anti-VEGF drugs is significant.

Hata M et al. demonstrated that PCV with a pachychoroid phenotype responds more effectively to the PDT, particularly regarding BCVA and the required number of treatments, compared to PCV with a non-pachychoroid phenotype [37]. Furthermore, PDT is the most effective treatment for central serous chorioretinopathy, one of the representative diseases of pachychoroid-spectrum disorders [38]. The choroidal blood flow overload is indicated as a crucial pathogenetic factor in pachychoroid-spectrum disorders [39, 40], and PDT is suggested to alleviate stasis throughout the choroid, extending beyond the irradiated areas [41]. These findings correspond with the higher observed efficacy of PDT in treating PCV with a pachychoroid phenotype.

In the present study, cases that did not require additional treatment were significantly older than those that required further treatment. Previous reports have indicated that PDT can decrease choroidal blood flow, potentially alleviating choroidal vascular stasis [41, 42]. Furthermore, choroidal blood flow has been reported to decline with increasing age [43]. In our study, the group that responded more effectively to treatment was relatively older. This may have resulted in lower baseline choroidal blood flow in this group. Consequently, the decrease in blood flow induced by PDT could have been sufficient to achieve the therapeutic effect.

Additionally, while the difference was not statistically significant, 100% of patients in the group not requiring further treatment had CVH, compared to 57.9% of patients in the group that required additional treatment. Previous reports indicate that AMD with pachychoroid features tends to be more responsive to PDT, and has a higher prevalence of CVH compared to non-pachychoroid AMD [37, 44]. Therefore, the group that did not require additional treatment in the current study may have had more PCV with pachychoroid characteristics than the group that did require additional treatment.

However, the beneficial aspects of PDT and the risks need to be considered in the choice of treatment. In our study, the incidence of ocular complications was 8.3% in the PDT group and 18.2% in the brolucizumab group. PDT has been associated with potential vision loss due to SRH and retinal damage [27, 45], and the incidence of ocular adverse events in combination therapy was reported as 1.7% in the EVEREST II study, 3.1% in the PLANET study, and 2.8% in the FUJISAN study [7, 22, 46]. Furthermore, brolucizumab has been associated with reports of intraocular inflammation, retinal vasculitis, and retinal vascular occlusion [36, 47]. Identifying biomarkers that can predict both the efficacy and the likelihood of complications of these treatments is crucial for targeting PDT in cases where it can be more effective and safer.

A VEGF plays a crucial role in the activity of macular neovascularization in nAMD, making anti-VEGF agents essential for nAMD treatment [48]. However, some nAMD patients do not respond well to anti-VEGF agents, particularly in terms of progressive loss of response to anti-VEGF therapy, a phenomenon known as tachyphylaxis [49]. A possible mechanism of anti-VEGF agent resistance is the activation of a VEGF-independent angiogenic signaling pathway by anti-VEGF agents [50]. Since the nAMD patients included in this study experienced recurrence or even worsening within a short period after anti-VEGF treatment, these patients might be VEGF-independent or partially VEGF-independent.

While PDT is thought to act on macular neovascularization through selective occlusion of macular neovascularization and choriocapillaris [51]. Therefore, if blocking the pathway with one agent becomes ineffective, PDT, which physically occludes the macular neovascularization, or switching to another agent with a different mechanism of action might be effective. In fact, PDT was highly effective in approximately 20% of the cases where it was used as a second-line treatment in this study. Recently, faricimab, an angiopoietin-2 inhibitor, has been introduced for nAMD treatment, and its efficacy in treating refractory nAMD warrants further investigation. [52]

This study's limitations include its retrospective nature and the relatively small number of cases, and the selection and timing of treatment were at the discretion of the individual physicians. To minimize bias in choosing between PDT and brolucizumab, the study design limited PDT cases to the period before brolucizumab was available. Additionally, statistical methods were employed to adjust for complications and other factors influencing treatment outcomes. Indeed, the current adjusted OR for additional treatment-free cases may lean towards the null due to adjustments for social factors like lifestyle, income, education, and treatment responsiveness, which are inherently unmeasurable. This study, employing E-value analysis, determined that the estimated OR would only be nullified if confounding factors had an association, with an odds ratio (OR) of 23.44, to both the treatment choice and its outcome. In addition, the probability of such a scenario is considered low. Therefore, attributing the relationship between PDT and the occurrence of additional treatment-free cases solely to bias is challenging. Moreover, in this study, we did not evaluate factors influencing treatment outcomes, including dye leakage and occlusion of polypoidal lesions, due to the absence of cases where FA and ICGA were conducted following second-line treatment. Future research demands a focus on investigating the effect of PDT and brolucizumab as second-line treatments for PCV in interventional studies. Furthermore, as a second-line treatment for PCV, the differences in efficacy and safety between PDT monotherapy and combination therapy with PDT remain unclear. Future studies should compare treatment outcomes between these two approaches.

## Conclusions

In conclusion, PDT and brolucizumab demonstrated no significant differences in 12-month visual and anatomical outcomes when used as second-line treatments for PCV. Moreover, some cases of PCV resistant to anti-VEGF drugs may not need further treatment for a year when treated with PDT. However, the choice of treatment should consider the risk of potential complications.

### Supplementary Information

**Additional file 1.** Causal directed acyclic graph (DAG) for assessing the association between treatment selection and outcomes.

**Additional file 2: Table S1.** Comparison of best-corrected visual acuity between brolucizmab andphotodynamic therapy groups at each time point.

**Additional file 3: Table S2.** Logistic regression analyses with cases that did not require additional treatments followinga second-line treatment as a dependent variable.

## Data Availability

The datasets generated and/or analyzed during the current study are not publicly available due to making the data release was not included in the research plan and was not communicated to the patients but are available from the corresponding author on reasonable request.

## Abbreviations

nAMD: Neovascular age-related macular degeneration
PCV: Polypoidal choroidal vasculopathy
VEGF: Vascular endothelial growth factor
PDT: Photodynamic therapy
CFP: Color fundus photograph
PED: Pigment epithelial detachment
ICGA: Indocyanine green angiography
SD-OCT: Spectral domain-optical coherence tomography
SRH: Subretinal hemorrhage
BCVA: Best-corrected visual acuity
CMT: Central macular thickness
SFCT: Subfoveal choroidal thickness
SRF: Subretinal fluid
IRF: Intraretinal fluid
