# Supplementary material for: Evaluating photodynamic therapy versus brolucizumab as a second-line treatment for polypoidal choroidal vasculopathy
Source: Int J Retina Vitreous. 2024 Apr 8;10:32. doi: 10.1186/s40942-024-00553-5 (PMC11000321; doi:10.1186/s40942-024-00553-5)
Supplement: Supplementary file 1 — Additional file 1. Causal directed acyclic graph (DAG) for assessing the association between treatment selection and outcomes. [file 40942_2024_553_MOESM1_ESM.pdf]

Figure S1. Causal directed acyclic graph (DAG) for assessing the association between treatment selection and outcomes

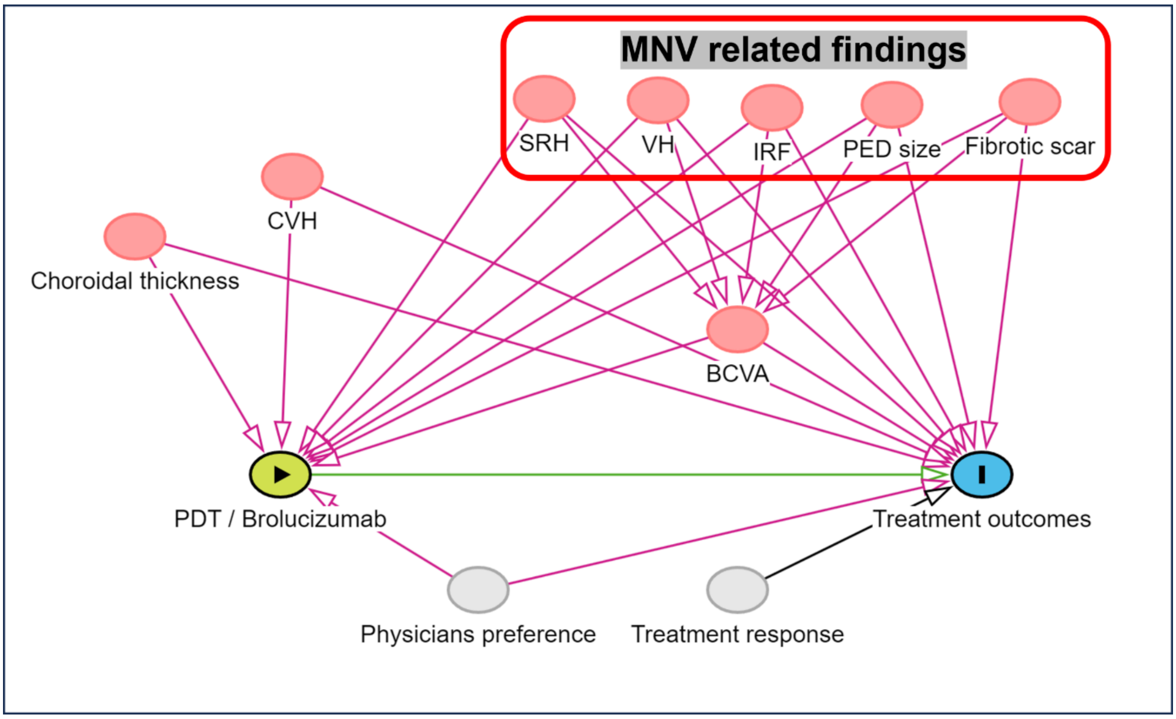

Red circles represent adjusted potential confounders. Physician preference is a potential confounding factor, but it could not be adjusted for due to the retrospective nature of the study. **Abbreviations**: **MNV**, macular neovascularization; **SRH**, subretinal hemorrhage; **VH**, vitreous hemorrhage; **IRF**, intraretinal fluid; **PED**, pigment epithelial detachment; **CVH**, choroidal vascular hyperpermeability; **BCVA**, best-corrected visual acuity; **PDT**, photodynamic therapy.
