# Supplementary material for: Evaluating photodynamic therapy versus brolucizumab as a second-line treatment for polypoidal choroidal vasculopathy
Source: Int J Retina Vitreous. 2024 Apr 8;10:32. doi: 10.1186/s40942-024-00553-5 (PMC11000321; doi:10.1186/s40942-024-00553-5)
Supplement: Supplementary file 2 — Additional file 2: Table S1. Comparison of best-corrected visual acuity between brolucizmab andphotodynamic therapy groups at each time point. [file 40942_2024_553_MOESM2_ESM.pdf]

Table S1. Comparison of best-corrected visual acuity between brolucizmab and photodynamic therapy groups at each time point

BCVA at each time point (logMAR)	Brolucizmab, N = 22	Photodynamic therapy, N = 24	P-values†
Baseline	0.48 ± 0.52; 0.35	0.30 ± 0.33; 0.30	0.314
1M	0.47 ± 0.44; 0.35	0.34 ± 0.35; 0.22	0.335
P-values*	0.906	0.054	
2M	0.35 ± 0.32; 0.30	0.25 ± 0.32; 0.15	0.289
P-values*	0.650	0.917	
3M	0.36 ± 0.33; 0.30	0.37 ± 0.40; 0.22	0.817
P-values*	0.059	0.177	
6M	0.39 ± 0.33; 0.40	0.28 ± 0.35; 0.15	0.186
P-values*	0.836	0.616	
9M	0.36 ± 0.30; 0.30	0.34 ± 0.37; 0.30	0.687
P-values*	0.258	0.410	
12M	0.49 ± 0.47; 0.35	0.34 ± 0.39; 0.30	0.236
P-values*	0.465	0.330	

The mean ± standard deviation; median was used for continuous variables. †These P-values were derived from comparisons between the brolucizmab and photodynamic therapy groups using the Mann-Whitney U test. *P-values for these comparisons were obtained by evaluating the BCVA at each time point against the baseline using the Wilcoxon signed-rank test. **Abbreviations**: BCVA, best-corrected visual acuity.
