# Supplementary material for: Evaluating photodynamic therapy versus brolucizumab as a second-line treatment for polypoidal choroidal vasculopathy
Source: Int J Retina Vitreous. 2024 Apr 8;10:32. doi: 10.1186/s40942-024-00553-5 (PMC11000321; doi:10.1186/s40942-024-00553-5)
Supplement: Supplementary file 3 — Additional file 3: Table S2. Logistic regression analyses with cases that did not require additional treatments followinga second-line treatment as a dependent variable. [file 40942_2024_553_MOESM3_ESM.pdf]

Table S2. Logistic regression analyses with cases that did not require additional treatments following a second-line treatment as a dependent variable

Characteristics	Regression coefficients* 95%CI	OR 95%CI	P-values	Partial regression coefficients†	Adjusted OR	P-values
PDT ref. Brolucizmab	2.54<br>0.26–7.44	12.69<br>1.30–1706.23	0.025	2.48<br>0.35–7.64	11.98<br>1.42–2070.07	0.019
SRH	-0.56<br>-2.93–1.24	0.57<br>0.05–3.46	0.591	-1.31<br>-4.61–1.32	0.27<br>0.01–3.76	0.344
Fibrotic scar	0.00<br>-1.88–1.74	1.00<br>0.15–5.72	0.999	0.63<br>-2.09–3.87	1.88<br>0.12–47.81	0.648
BCVA (logMAR)	0.05<br>-2.45–1.83	1.05<br>0.09–6.26	0.962	-0.11<br>-4.84–4.56	0.90<br>0.01–95.94	0.961
IRF	0.67<br>-1.07–2.56	1.96<br>0.34–12.88	0.443	1.41<br>-0.64–4.26	4.09<br>0.53–70.77	0.184
CVH	1.54<br>-0.76–6.44	4.66<br>0.46–629.26	0.222	1.90<br>-0.91–7.89	6.70<br>0.40–2668.36	0.220
CCT	0.00<br>-0.01–0.01	0.72<br>0.99–1.01	0.716	0.00<br>-0.02–0.02	1.00<br>0.98–1.02	0.898
Maximum PED size	0.00<br>-0.00–0.00	1.00<br>1.00–1.00	0.383	0.00<br>-0.00–0.10	1.00<br>1.00–1.01	0.170

These regression coefficients are calculated by logistic regression analysis using Firth's bias reduction method*, multivariable logistic regression analysis (type of a second-line treatment, SRH, Fibrotic scar, BCVA, IRF, CVH, CCT) †. **Abbreviations**: PDT, photodynamic therapy; SRH, subretinal hemorrhage; BCVA, best-corrected visual acuity; IRF, intraretinal fluid; CVH, choroidal vascular hyperpermeability; CCT, central choroidal thickness; PED, pigment epithelial detachmen.
